# Characteristics of Walk-In Patients and Related Factors in a Dental University Hospital

**DOI:** 10.3390/healthcare10081469

**Published:** 2022-08-04

**Authors:** Won Hwa Jeon, Bomgyeol Kim, Do Hee Kim, Sang Gyu Lee, Suk-Yong Jang, Tae Hyun Kim

**Affiliations:** 1 Department of Conservative Dentistry, Kyung Hee University Dental Hospital, 23 Kyungheedae-ro, Seoul 02447, Korea; 2 Department of Public Health, Yonsei University, 50-1 Yonsei-ro, Seoul 03722, Korea; 3 Department of Preventive Medicine, College of Medicine, Yonsei University, 50-1 Yonsei-ro, Seoul 03722, Korea; 4 Department of Healthcare Management, Graduate School of Public Health, Yonsei University, 50-1 Yonsei-ro, Seoul 03722, Korea

**Keywords:** appointment scheduling, dental clinic, outpatient, walk-in patients

## Abstract

This study aimed to identify the characteristics of walk-in patients who visited a dental university hospital more than once (returning patient). The factors affecting walk-in visits were analyzed in relation to demographic, appointment, and treatment characteristics among 146,567 cases treated between 1 March 2019 and 29 February 2020. Multiple logistic regression was used to assess factors influencing walk-in visits. The walk-in rate was 14.1%. The likelihood of walk-in visits was higher in men, patients aged 20–39 years, patients residing in Seoul, and hospital employees or their family members. Walk-in visits were more likely to take place from 8:00 to 9:59 and on Saturdays and Mondays. The walk-in odds ratios differed depending on the treatment department and diagnosis. Return patients had higher odds of walk-in visits for treatments not covered by insurance. The probability of being a walk-in patient was lower among patients who also received treatment in other departments on the same day than among those who did not. These results indicate that each examined factor has a predictable pattern. The findings also suggest a relatively high percentage of walk-in cases in dental university hospitals and that walk-in patients differ in their characteristics from patients with appointments.

## 1. Introduction

Currently, most companies adopt an in-advance reservation system to provide timely services to customers [1]. In addition, walk-in customers are often received for various reasons [2]. A company benefits from offering services to walk-in customers in various ways, such as increasing corporate profits, expanding customer pools, and building a positive business reputation. Banks allow for same-day walk-in visits. Hotels with vacant rooms provide services to walk-in customers. Similarly, restaurants rely on same-day visits for word-of-mouth marketing, whereas the beauty industry allows for same-day visits to secure regular customers. Repair centers offer their services to same-day walk-in clients to appeal to more customers [2].

However, same-day walk-in customers may disrupt daily business operations as such clients turn up without prior notice and affect the services provided to customers who made reservations in advance [3,4]. In the medical industry, a considerable conflict between reservations and walk-ins has been acknowledged [2]. This is particularly important because providing timely medical services to patients is crucial [4]. In general, because hospital hours are fully booked in advance, unexpected arrivals of walk-in patients lead to overbooking and unnecessary increases in waiting time [5]. Accordingly, same-day appointments may be difficult to accommodate in an already overbooked service [6]. However, most hospitals in Korea accept a considerable number of walk-in patients.

Patients without appointments at dental clinics can receive treatment on the same day. However, there are no time slots specifically assigned to walk-in patients, as almost all hospital services require a prior appointment. This forces medical institutions to treat walk-in patients in reserved slots of patients who did not show up or canceled their appointments or to adjust the treatment scope of patients with appointments [7]. Furthermore, treatments in dental departments require a relatively long time compared to those in other outpatient departments, and because of the workspace and space limitations, delays in treatment and an increase in waiting time are inevitable for walk-in patients. Considering such particularities, dental hospitals would benefit from a more efficient outpatient appointment system than other hospital departments [7]. Therefore, walk-in patient management in dental hospitals is emerging as a critical issue that must be addressed.

Efforts have been made to investigate and manage the current status and trends of walk-in patients in overseas hospitals. In the United States, walk-in patients in primary care account for 10–60% of daily hospital visitors [8,9,10]. An average of 72% of walk-in patients present at medical institutions in Taiwan [11]. In China, walk-in treatments are common, representing a high proportion of the total patients [12]. Studies in Germany have reported that all patients are required to book an appointment in advance, and yet 5% of all patients are walk-in patients [13]. In Japan, more than 20% of outpatients are walk-in patients [3].

Overseas stakeholders have addressed various walk-in-related factors that reduce care efficiency and increase patient waiting times. By contrast, data on the profile of walk-in patients attending dental hospitals in Korea and the activities of these units are scarce. Knowledge on this topic is necessary not only to optimize car, but also as the first step in ensuring the quality of medical services. Thus, this study aimed to assess the current status of walk-in patients and patients with appointments at dental departments of university hospitals in Korea and to analyze the characteristics and trends of walk-in patients.

## 2. Materials and Methods

### 2.1. Data Collection

This study included patients treated in the outpatient department of a dental university hospital located in Seoul. A total of 181,365 outpatients were registered for the one-year period from 1 March 2019 to 29 February 2020.

The appointment status was based on the criteria of “visits of walk-in patients” and “visits of patients with appointments,” whereas “requests,” which include consultation requests from other departments, were excluded as they represent an additional form of patient reception. This study targeted the eight major departments that provide an appointment-based treatment: oral medicine, oral and maxillofacial surgery, orthodontics, endodontics, prosthodontics, pedodontics, implant center, and periodontology. Data from other centers and clinics were excluded.

A new visit to the department refers to a patient being treated for the first time in a given department. A returning visit refers to the visit of a patient who had received treatment at least once in a specific department. This study included the returning patients visiting the hospital without an appointment from the perspective of healthcare providers (hospital management). Thus, new patients were excluded, and only returning patients were examined. A total of 146,567 patients were selected for the final data analysis.

### 2.2. Measures

#### 2.2.1. Dependent Variable

The dependent variable was whether the patient was a walk-in patient, which was recorded either as “walk-in (patient) = 1” or “outpatient appointment (patient) = 0.” Walk-in patients were defined as patients who came to the hospital without a prior appointment or who made an appointment on the same day by phone.

#### 2.2.2. Independent Variables

- Demographic characteristics

The sociodemographic characteristics of the study participants recorded were sex, age, patient’s residence, and being a hospital employee or a family member of a hospital employee. The participants were categorized based on sex as male and female, and based on age (from 0 to 70 years), they were categorized into seven groups of 10-year increments (≤19, 20–29, 30–39, 40–49, 50–59, 60–69, and ≥70 years). The patients’ place of residence was classified into northeast Seoul, southeast Seoul, northwest Seoul, southwest Seoul, Gyeonggi-do, and other regions, according to the administrative regional division of Korea. The status of being a hospital employee or a family member of a hospital employee referred to the employees’ families registered in the Human Resources Department, including the faculty members themselves, and was distinguished from the status of not being related to hospital employees.

- Appointment-related characteristics

The outpatient hours of the target hospital were from 9:00 to 12:00 and from 13:30 to 17:30 on weekdays (Monday–Friday). On Wednesdays, nighttime treatment was provided with additional operations from 18:00 to 20:00. On Saturdays, outpatient treatments were conducted only in the morning from 9:00 to 12:30. Considering the above, this study categorized the treatment hours into five slots: “8:00–9:59,” “10:00–12:29,” “12:30–14:59,” “15:00–17:29,” and “17:30 and thereafter.” Treatment was provided from Monday to Saturday at regular hours, and treatment days were categorized by month from January to December.

- Treatment-related characteristics

To identify relationships among walk-in patients, departmental characteristics, and symptoms, we used the following categories. Based on the insurance type, treatments were divided into covered and noncovered treatments. Treatments in this study were classified as “orthodontic treatment,” “oral medicine treatment,” “oral surgery treatment,” “endodontic treatment,” “neurotherapy,” “periodontal treatment,” “traumatic treatment,” “prosthetic treatment,” and “other,” based on the main diagnosis of the patient according to the KCD-7 code (Korean version of the ICD-10) [14]. In the case of a treatment received from another department, it was classified as “no treatment from another department” or “treatment from another department” to confirm whether the patient simultaneously received treatment from a medical university hospital having dentistry and oriental medicine departments on the same day.

### 2.3. Statistical Analysis

A frequency analysis was conducted to compare the demographic characteristics, appointment-related characteristics, treatment types, and data distribution of the study population. The results of this analysis are presented as frequencies and percentages. The chi-square test was performed to verify differences in the characteristics of the study participants depending on their status as walk-in patients. In addition, multiple logistic regression analysis was conducted to determine the factors that influence walk-in visits. The unit used for this analysis was the outpatient visit of each patient, not the individual patient. Thus, if the same patient visited the hospital multiple times, their status may have been different for each visit. The assumption of independent events in the observation period could be violated if the same patient visited the hospital multiple times. To control for this, we conducted repeated measures (using the “repeated subject” option) in the generalized estimating equation.

### 2.4. Ethical Issues

This study adhered to the Declaration of Helsinki and was reviewed by the Institutional Review Board of Kyung Hee University Dental Hospital (protocol code KH-DT20037). The requirement for written informed consent from patients was waived because the study involved secondary data analyses using de-identified data.

## 3. Results

### 3.1. Characteristics of Study Participants

The Table 1 shows the comparison of characteristics between walk-in and appointment visits. Among all participants, walk-in visits were registered for 13.3% of female patients and 15.1% of male patients, indicating that men were more likely to have walk-in visits. Regarding patient age, 12.3% of patients aged 19 years and younger were walk-in patients, whereas the proportion of walk-in patients was 19.8% among those aged 20–29 years, 14.7% among patients aged 30–39 years, 11.8% among patients aged 40–49 years, 13.9% in those aged 50–59 years, 11.7% in patients aged 60–69 years, and 14.2% in patients aged 70 years or older; hence, patients aged 20–29 years had the highest percentage of walk-in visits. An analysis by region revealed that the percentage of walk-in patients was 13.4% in northeast Seoul, 19.0% in southeast Seoul, 21.3% in northwest Seoul, 24.6% in southwest Seoul, 12.3% in Gyeonggi-do Province, and 18.0% in other cities. Thus, the percentages of walk-in patients were higher in the Seoul region, where the research hospitals are located.

The highest percentages of walk-ins occurred in the time slot of 8:00–9:59, corresponding to the first time slot for visits. Regarding the day of the week, the highest percentages were recorded on Saturday (19.6%) and Monday (15.8%). December (16.2%) had the highest percentage of walk-in visits, followed by June (15.8%) and May (14.9%).

The number of walk-in visits was higher in the noncovered treatment group (16.5%). In terms of departments, the rate of walk-in patients was as follows: orthodontics, 5.9%; oral medicine, 11.8%; oral and maxillofacial surgery, 20.6%; conservative dentistry, 16.0%; prosthodontics, 18.7%; pedodontics, 14.7%; periodontology, 14.4%; and implant center, 2.5%. Hence, the oral and maxillofacial surgery department reported the highest percentage of walk-ins, followed by the prosthodontics department.

Further, 8.1% of patients with orthodontic treatment were walk-in patients, whereas 22.0% of patients with oral medicine and orofacial pain treatment, 11.3% of patients who underwent oral surgery, 18.7% patients who received conservative treatment, 11.3% patients who received nerve treatment, 21.6% patients who received periodontal treatment, 20.0% patients who received treatment for trauma, 14.7% patients who underwent prosthodontic treatment, and 17.9% of patients who received other treatments were walk-in patients. A high percentage of walk-in visits was also observed among patients receiving no treatment in other departments on the day of dental treatment (14.3%).

### 3.2. Factors Related to Walk-In Visits

Table 2 shows the results of the multiple logistic regression identifying the factors related to walk-in visits. Compared to women, men were more likely to have walk-in visits (odds ratio (OR) = 1.12). Patients aged 20–29 (OR = 1.71) and 30–39 years (OR = 1.23) were more likely to have walk-in visits than those aged 19 years or younger, whereas patients aged 60–69 (OR = 0.86) were less likely to have walk-in visits. Regarding the place of residence, patients residing in southeast Seoul (OR = 1.48), northwest Seoul (OR = 1.60), southwest Seoul (OR = 1.78), and other Si or Do provinces (OR = 1.28) were more likely to have walk-in visits, and patients residing in Gyeonggi-do (OR = 0.92) were less likely to have walk-in visits. Further, the likelihood of having walk-in visits was higher among employees or their family members (OR = 1.23).

Compared to the time slot of 8:00–9:59, time slots for treatments between 10:00 and 12:29 (OR = 0.65), between 15:00 and 17:29 (OR = 0.59), and after 17:30 (OR = 0.55) were less likely to have walk-in visits. Walk-in visits were more likely to occur on Saturday than on Monday (OR = 1.19), and the probability was lower on all other days of the week. Regarding treatment months, compared to January, walk-in visits were more likely to occur in December (OR = 1.19), November (OR = 1.12), June (OR = 1.12), May (OR = 1.11), and March (OR = 1.09).

Compared to covered treatment, noncovered treatment (OR = 4.07) was more likely to be the reason for walk-in visits. Compared to the orthodontics department, the departments of oral medicine (OR = 6.73), pediatric dentistry (OR = 6.41), oral and maxillofacial surgery (OR = 6.27), conservative dentistry (OR = 4.05), periodontology (OR = 3.01), and prosthetics (OR = 1.60) had a higher probability of walk-in visits. However, the probability for dental implant centers was low (OR = 0.53). Regarding the diagnosis, the probability of walk-in visits was higher for periodontal treatment (OR = 2.66), trauma treatment (OR = 2.55), oral medicine treatment (OR = 2.45), prosthetic treatment (OR = 2.01), conservative treatment (OR = 1.77), nerve treatment (OR = 1.51), oral surgery (OR = 1.50), and other treatments (OR = 1.89) than for malocclusion periodontal treatment. Patients who received treatments in the dental and other departments on the same day were less likely to be walk-in patients than those who did not receive treatments in two or more departments (OR = 0.89).

## 4. Discussion

Appointment systems in many service facilities, especially hospitals, are used to manage walk-ins in addition to scheduled arrivals [15]. Walk-in patients are usually assigned the lowest priority depending on the occurrence of “no shows”. This may disrupt the original schedule and affect the performance of the appointment system; for instance, the waiting time of scheduled patients. This study is an attempt to gain insights into the appropriate management of patients in the presence of walk-ins.

The main purpose of this study was to identify factors related to outpatient walk-in visits to better manage walk-in visits and facilitate the efficient operation of hospital treatments. The rate of walk-in visits by study participants and return patients was 14.1%. This walk-in rate was lower than the 20.0% reported by Morikawa and Takahashi in Japan and slightly higher than the 5.0% reported by Riedl et al. (2009) in Germany [3,13]. However, direct comparisons between the above results and the findings of this study are difficult owing to the differences in the treatment environment. However, the walk-in rate of returning patients was judged to be relatively high, thus emphasizing the need for effective management.

Factors associated with walk-in patients in previous studies were also identified as significant factors in this study. The factors influencing walk-ins found in this study can be summarized as follows in comparison with existing studies. First, men were more likely to have walk-in visits than women. This result was similar to the findings of Riedl et al. (2018) and Geelan-Hansen et al. (2021), who observed a higher incidence of walk-in by men than by women [5,16]. This result is consistent with the view that men visit the dental emergency department more often than women owing to their higher risk of trauma while practicing outdoor activities such as sports or driving. In addition, the range of physical activity is relatively wide among men, and they tend to endure pain for longer periods of time before seeking assistance, which may lead to acute conditions and urgent visits to the hospital [17].

Second, patients in the 20–29 and 30–39 age groups were more likely to visit the hospital without an appointment, and this result was similar to the high rate of 19–35-year-old walk-in patients observed by Geelan-Hansen et al. (2021) [16]. Moreover, this result is also similar the finding of Riedl et al. (2018), who reported a higher probability of walk-in patients at younger ages [5]. As such, it can be presumed that these results stem from issues in time schedules due to school and work activities, as well as a lack of awareness about appointment systems.

Third, employees or their family members were more likely to visit the hospital for walk-in visits. This result likely reflects their better access to treatment compared to that of the general patient population as the employees are linked to the hospital, which allows them and their families to receive treatment quickly, facilitating the walk-in visits in this group.

Fourth, walk-in visits were most likely to occur during the first time slot of treatment, between 8:00 and 9:59. Patients may have the expectation of receiving treatment if they arrive early at the hospital and wait even without an appointment, and their prior experiences of walk-in may have played a role in creating this expectation. Regarding the days of treatment, the probability of walk-in visits was higher on Saturdays and Mondays. Cayirli and Gunes also observed the highest number of walk-in patients on Mondays, which is in line with the results of this study [18]. Further, it may be reasonable to assume that walk-in patients are more frequent on Mondays, when they seek to resolve health issues that emerged over the weekend. On the other hand, patients seeking treatment on Saturdays likely do so because of time constraints due to work- and school-related schedules.

Fifth, regarding the insurance type, there was a high probability of walk-in visits for noncovered treatments. This result may be attributable to problems such as fallen prosthesis, dental restorations, and orthodontic treatments, which are common among relatively young patients and are not covered by insurance. Nevertheless, it is difficult to establish a clear reason for this, and thus further research is necessary.

Furthermore, we found the significant differences in the trend of walk-in patient visits across the departments and diagnosis groups. Regarding the treatment-related diagnosis, compared to orthodontic treatment, the probability of walk-in visits was higher for all other disease categories. In particular, the likelihood of periodontal and trauma treatment was particularly high. Considering that the KCD-7 code for periodontal treatment covers a broad range of disorders in dental care, a detailed review is needed in future studies. In addition, patients are assigned to different departments depending on their conditions or age groups (pediatrics, the disabled, orthodontics, and geriatrics), and the structure of the respective hospitals or clinics, such as the existence of specialized departments. In general, patients visit the dental hospital for four reasons: orofacial pain, oral functional issues, impaired orofacial appearance, and psychosocial issues [19]. In such a case, children were usually assigned to the children’s department regardless of their specific issues. Therefore, the study results should be interpreted accordingly with the caution since departments utilized varying appointment systems.

This study had several limitations. First, it was conducted based on the one-year outpatient data from a dental university hospital in Seoul. This limits the applicability of the study results to all dental university hospitals. Future research should expand the scope to dental university hospitals across multiple regions. Second, this study did not include some of the variables due to the unavailability of hospital data such as the socioeconomic status of patients. Further study would be recommended to identify the walk-in patient visits according to the socioeconomic status of the patients by using the administrative data such as health insurance data, whereas hospital data are not possible. Third, this was a cross-sectional study using the medical records of the previous year, which limits the interpretation of causal relationships between independent and dependent variables. Finally, the dental disease codes of the KCD-7 cover a broad range of disorders treated in dental university hospitals. This study categorized diseases into nine groups with reference to existing studies, but caution is required when interpreting the data because one disease type can relate to a range of treatments. Despite these limitations, since only a few prior studies have examined walk-in patient visits in Korea, this study is meaningful in that it is the first to provide data on walk-in patient visits in dental university hospitals.

## 5. Conclusions

Healthcare providers must make contingency plans for unpredictable demands. Returning patients typically prefer appointment-based treatments. However, 14.1% of them are walk-in patients. Considering care quality and patient satisfaction, it is necessary to optimize the management of walk-in patients.

To this end, hospital managers should consider introducing advanced access scheduling, an appointment scheduling system that allows patients to seek and receive health care from their provider at the time of their choice. From a financial perspective, practice leaders and providers may be able to maximize patient volume and revenue through advanced access scheduling.

Similar to other interventions, this scheduling method must consider the characteristics of particular patient populations and practice settings. Based on the characteristics of walk-in patients identified in this study, partial adoption of advanced access scheduling may be considered for the first time slot (8:00–9:59) in departments such as pediatric dentistry or oral and maxillofacial surgery, which receive a high percentage of walk-in patients. The potential benefits are numerous, but further research is imperative to identify all effects as well as the best strategies for implementation.

## Figures and Tables

**Table 1:** Comparison between walk-in and appointment visits.

Variables	Total		Walk-In Visits		Appointment Visits		*p*
	N	%					
Total	146,567	100.0	20,666	14.1	125,901	85.9	
Sex							<0.0001
Male	81,453	55.6	10,883	13.3	70,620	86.7	
Female	65,114	44.4	9833	15.1	55,281	84.9	
Age (years)							<0.0001
≤19	36,851	25.1	4532	12.3	32,319	87.7	
20–29	24,874	17.0	4930	19.8	19,944	80.2	
30–39	12,254	8.4	1803	14.7	10,451	85.3	
40–49	12,655	8.6	1491	11.8	11,164	88.2	
50–59	19,047	13.0	2638	13.9	16,409	86.2	
60–69	21,273	14.5	2488	11.7	18,785	88.3	
≥70	19,613	13.4	2784	14.2	16,829	85.8	
Region							<0.0001
Northeast Seoul	89,316	60.9	11,950	13.4	77,366	86.6	<0.0001
Southeast Seoul	9013	6.2	1715	19.0	7298	81.0	
Northwest Seoul	4217	2.9	896	21.3	3321	78.8	
Southwest Seoul	2350	1.6	579	24.6	1771	75.4	
Gyeonggi regions	34,493	23.5	4233	12.3	30,260	87.7	
Other regions	7178	4.9	1293	18.0	5885	82.0	
Employee/family member							0.1939
No	140,503	95.9	19,776	14.1	120,727	85.9	
Yes	6064	4.1	890	14.7	5174	85.3	
Treatment hours							<0.0001
8:00–9:59	31,088	21.2	6157	19.8	24,931	80.2	
10:00–12:29	41,920	28.6	4541	10.8	37,379	89.2	
12:30–14:59	35,355	24.1	6421	18.2	28,934	81.8	
15:00–17:29	33,705	23.0	3139	9.3	30,566	90.7	
17:30 and thereafter	4499	3.1	408	9.1	4091	90.9	
Treatment days							<0.0001
Monday	28,603	19.5	4529	15.8	24,074	84.2	
Tuesday	26,612	18.2	3417	12.8	23,195	87.2	
Wednesday	32,653	22.3	3827	11.7	28,826	88.3	
Thursday	23,943	16.3	3507	14.7	20,436	85.4	
Friday	25,623	17.5	3592	14.0	22,031	86.0	
Saturday	9133	6.2	1794	19.6	7339	80.4	
Treatment months							<0.0001
January	12,114	8.3	1608	13.3	10,506	86.7	
February	10,050	6.9	1420	14.1	8630	85.9	
March	12,166	8.3	1758	14.5	10,408	85.6	
April	12,747	8.7	1791	14.1	10,956	86.0	
May	12,575	8.6	1869	14.9	10,706	85.1	
June	11,890	8.1	1875	15.8	10,015	84.2	
July	13,692	9.3	1782	13.0	11,910	87.0	
August	13,610	9.3	1645	12.1	11,965	87.9	
September	11,180	7.6	1452	13.0	9728	87.0	
October	13,085	8.9	1796	13.7	11,289	86.3	
November	11,989	8.2	1811	15.1	10,178	84.9	
December	11,469	7.8	1859	16.2	9610	83.8	
Insurance types							0.3997
Covered treatment	51,161	88.3	42,659	83.4	8502	16.6	
Noncovered treatment	6811	11.7	5651	83.0	1160	17.0	
Treatment department							<0.0001
Oral medicine	19,651	13.4	1155	5.9	18,496	94.1	
Oral and Maxillofacial surgery	18,955	12.9	2233	11.8	16,722	88.2	
Orthodontics	19,803	13.5	4087	20.6	15,716	79.4	
Endodontics	18,624	12.7	2986	16.0	15,638	84.0	
Prosthetics	19,100	13.0	3579	18.7	15,521	81.3	
Pediatric dentistry	21,888	14.9	3223	14.7	18,665	85.3	
Periodontology	22,584	15.4	3255	14.4	19,329	85.6	
Implant center	5962	4.1	148	2.5	5814	97.5	
Disease category							<0.0001
Orthodontic treatment	40,984	28.0	3308	8.1	37,676	91.9	
Oral medicine treatment	5217	3.6	1146	22.0	4071	78.0	
Oral surgery treatment	32,521	22.2	3683	11.3	28,838	88.7	
Endodontic treatment	18,118	12.4	3380	18.7	14,738	81.3	
Neurotherapy	12,440	8.5	1404	11.3	11,036	88.7	
Periodontal treatment	28,552	19.5	6178	21.6	22,374	78.4	
Trauma treatment	3480	2.4	695	20.0	2785	80.0	
Prosthetic treatment	2140	1.5	315	14.7	1825	85.3	
Other	3115	2.1	557	17.9	2558	82.1	
Treatment received from another department							<0.0001
No	124,604	85.0	17,840	14.3	106,764	85.7	
Yes	21,963	15.0	2826	12.9	19,137	87.1	

**Table 2:** Multiple logistic regression of factors associated with walk-in visits.

Variables	OR	95% CI		*p*
Sex				
Male	ref			
Female	1.12	1.07	1.17	<0.0001
Age (years)				
≤19	ref			
20–29	1.71	1.56	1.87	<0.0001
30–39	1.23	1.11	1.37	0.0002
40–49	0.91	0.81	1.03	0.1230
50–59	0.93	0.83	1.04	0.1917
60–69	0.86	0.77	0.97	0.0122
≥70	1.09	0.97	1.23	0.1330
Region				
Northeast Seoul	ref			
Southeast Seoul	1.48	1.36	1.61	<0.0001
Northwest Seoul	1.60	1.42	1.82	<0.0001
Southwest Seoul	1.78	1.50	2.10	<0.0001
Gyeonggi regions	0.92	0.87	0.97	0.0027
Other regions	1.28	1.15	1.43	<0.0001
Employee/family member				
No	ref			
Yes	1.23	1.10	1.37	0.0002
Treatment hours				
8:00–9:59	ref			
10:00–12:29	0.65	0.63	0.68	<0.0001
12:30–14:59	1.00	0.96	1.05	0.9624
15:00–17:29	0.59	0.56	0.62	<0.0001
17:30 and thereafter	0.55	0.49	0.61	<0.0001
Treatment days				
Monday	ref			
Tuesday	0.86	0.82	0.90	<0.0001
Wednesday	0.76	0.73	0.80	<0.0001
Thursday	0.84	0.80	0.88	<0.0001
Friday	0.84	0.80	0.89	<0.0001
Saturday	1.19	1.11	1.28	<0.0001
Treatment months				
January	ref			
February	1.04	0.97	1.12	0.2291
March	1.09	1.02	1.17	0.0118
April	1.03	0.96	1.10	0.4641
May	1.11	1.04	1.18	0.0029
June	1.12	1.05	1.20	0.0006
July	1.03	0.96	1.10	0.4195
August	0.98	0.92	1.05	0.5402
September	1.04	0.97	1.12	0.2417
October	1.02	0.96	1.09	0.5084
November	1.12	1.04	1.19	0.0012
December	1.19	1.12	1.27	<0.0001
Insurance types				
Covered treatment	ref			
Noncovered treatment	4.07	3.71	4.45	<0.0001
Treatment department				
Oral Medicine	ref			
Oral and Maxillofacial Surgery	6.73	5.91	7.66	<0.0001
Orthodontics	6.27	5.46	7.19	<0.0001
Endodontics	4.05	3.48	4.70	<0.0001
Prosthetics	1.60	1.40	1.83	<0.0001
Pediatric Dentistry	6.41	5.48	7.50	<0.0001
Periodontology	3.01	2.61	3.48	<0.0001
Implant Center	0.53	0.45	0.64	<0.0001
Disease category				
Orthodontic treatment	ref			
Oral medicine treatment	2.45	2.17	2.77	<0.0001
Oral surgery treatment	1.50	1.35	1.67	<0.0001
Endodontic treatment	1.77	1.58	1.98	<0.0001
Neurotherapy	1.51	1.32	1.73	<0.0001
Periodontal treatment	2.66	2.38	2.98	<0.0001
Trauma treatment	2.55	2.21	2.94	<0.0001
Prosthetic treatment	2.01	1.67	2.42	<0.0001
Other	1.89	1.64	2.19	<0.0001
Treatment received from another department				
No	ref			
Yes	0.89	0.85	0.93	<0.0001

Abbreviations: OR, odds ratio; CI, confidence interval; ref, reference.

## Data Availability

The data presented in this study are available on request from the corresponding author.
